# Particulate Matter 2.5 Induced Developmental Cardiotoxicity in Chicken Embryo and Hatchling

**DOI:** 10.3389/fphar.2020.00841

**Published:** 2020-06-05

**Authors:** Qixiao Jiang, Chao Zhang, Shen Chen, Limei Shi, Dao Chuan Li, Na Lv, Lianhua Cui, Yanxia Chen, Yuxin Zheng

**Affiliations:** ^1^Department of Toxicology, School of Public Health, Qingdao University, Qingdao, China; ^2^Department of Toxicology, School of Public Health, Sun Yat-sen University, Guangzhou, China; ^3^Department of Pharmacology, School of Pharmacy, Qingdao University, Qingdao, China; ^4^Department of Occupational Diseases, Occupational Disease Center, Qingdao Central Hospital, Qingdao, China

**Keywords:** particulate matter 2.5, developmental toxicity, chicken embryo, nuclear factor kappa-light-chain-enhancer of activated B cells, inducible nitric oxide synthase, matrix metallopeptidase 9

## Abstract

Particulate matter poses health risk to developing organisms. To investigate particulate matters with a diameter smaller than 2.5 um (PM2.5)-induced developmental cardiotoxicity, fertile chicken eggs were exposed to PM2.5 *via* air cell injection at doses of 0.05, 0.2, 0.5, 2, and 5 mg/egg kg. Morphological changes in the embryonic day four (ED4) and hatchling hearts were assessed with histological techniques. Heart rates of hatchling chickens were measured with electrocardiography. The protein expression levels of nuclear factor kappa-light-chain-enhancer of activated B cells p65 (NF-kb p65), inducible nitric oxide synthase (iNOS), and matrix metallopeptidase 9 (MMP9) were assessed with immunohistochemistry or western blotting in hatchling hearts. PM2.5 exposure elevated areas of heart in ED4 embryo, increased heart rate, and thickened right ventricular wall thickness in hatchling chickens. Immunohistochemistry revealed enhanced NF-kb p65 expression in hatchling hearts. Western blotting results indicated that both iNOS and MMP9 expression were enhanced by lower doses of PM2.5 exposure (0.2 and 0.5 mg/kg) but not 2 mg/kg. In summary, developmental exposure to PM2.5 induced developmental cardiotoxicity in chicken embryo and hatchling chickens, which is associated with NF-kb p65, iNOS, and MMP9.

## Introduction

Air pollution, especially particulate matter (PM) pollution, is a significant health risk factor for human beings (Yu G. et al., 2019). Generated from multiple resources: coal combustions, diesel exhausts, biomass burning, etc. (Cho et al., 2018; Leepe et al., 2019), PM pollution affects large area across the world (Giannadaki et al., 2016; Wang et al., 2019a). Some areas have relatively high levels, far beyond the healthy levels determined by the world health organization, posing a significant threat to human health (Giannadaki et al., 2016).

PM may be categorized according to the size of the particles. Among them, PM2.5 (particulate matters with a diameter smaller than 2.5 μm) attracts most attentions, since the small size of PM2.5 allows inhalation into deeper, even into systemic circulation, thus may induce detrimental health effects to the cardiovascular system, pulmonary system, etc. (Wilson and Suh, 1997; Xing et al., 2016; Chen et al., 2017). Among the negative outcomes associated with PM2.5 exposure, developmental toxicity is an important one, yet received relatively less attention. PM2.5 has been demonstrated to be capable of passing the placenta barrier (Teng et al., 2016) and expose developing embryo/fetus. Epidemiological data has associated in utero exposure to PM2.5 with developmental cardiotoxicity (Chen S. et al., 2019) and neurotoxicity (Lubczynska et al., 2017). However, a precise mode of toxicity and molecular mechanism for the developmental toxicity induced by PM2.5 in utero exposure is not yet available. Further investigation is urgently needed for better understanding of PM2.5-induced developmental toxicity and risk assessments.

Chicken embryo has been used as a development model for over 200 years (Vergara and Canto-Soler, 2012), which allows direct observation and manipulation of developing embryo. Moreover, chicken embryo is a closed system, allowing relatively accurate exposure to toxicants without the interference of maternal effects. In the current study, chicken embryo model was utilized to assess the potential developmental toxicities induced by PM2.5 exposure. PM2.5 was exposed to the chicken embryos *via* air cell injection, by which the PM2.5 was injected onto the air cell membrane without direct penetration into the egg. This method has been demonstrated to elicit comparable results to real-world exposure to environmental contaminants (Hoffman et al., 1996). Early (embryonic day four, ED4) and late development stages (hatchling chickens) were both investigated for a more comprehensive understanding of the developmental toxicity at different stages of development. For early development assessment, ED4 embryos were used, since the embryos are undergoing organogenesis at this stage, in which the vital organs such as brain, heart, and eyes are visible, but the organogenesis is still going on, and defects may be visible in response to extragenous disruptions (Vergara and Canto-Soler, 2012; Vancamp and Darras, 2017; Wittig and Munsterberg, 2019). For late development assessment, hatchling chickens were selected, since most organ systems finished development at this stage.

One major proposed mechanism of toxicity for the PM2.5 is the inflammatory response, which may contribute to the negative effects of PM2.5 in the cardiovascular system, pulmonary system, and nervous system (Zhao et al., 2013). Among the signaling molecules regulating inflammatory response, nuclear factor kappa-light-chain-enhancer of activated B cells (NF-kb) is a major one, which plays role in the production of cytokines, chemokines, and growth factors regulating the expression of genes involved in the immune and inflammatory responses (Frode-Saleh and Calixto, 2000). Its downstream signaling molecules include inducible nitric oxide synthase (iNOS) and matrix metallopeptidase 9 (MMP9). iNOS plays roles in the production of excessive amounts of nitric oxide (NO), while MMP9 participates in the degradation of the extracellular matrix during tissue remodeling (Lee et al., 2012; Wilmes et al., 2020). PM2.5 exposure has been associated with the stimulation of inflammatory reaction, but specific mechanism had not been fully elucidated yet. In the current study, expression levels of NF-kb, iNOS, and MMP9 were investigated in PM2.5-exposed hatchling chicken heart tissues, further revealing the roles of these molecules in PM2.5-induced developmental toxicity. This study adds to the knowledge base of PM2.5-exposure induced developmental toxicities and provided information about the potential molecular mechanisms.

## Materials and Methods

### Animals

Fertile chicken eggs were purchased from Linwen Trade Corp (Jining, China). Prior to incubation, the eggs were cleaned with 20% povidone iodine and carefully dipped dry with paper towel. Eggs were then candled in a dark room to mark the air cell with a pencil. The eggs were then weighed, numbered, and assigned evenly into treatment groups (vehicle control, 0.05, 0.2, 0.5, 2, and 5 mg/kg) according to the egg weight. Three to five alive embryonic day four (ED4) embryos per group were collected. Two batches of hatchling chickens were used, with a total of 8, 4, 8, 6, 8, and 4 hatchlings included for control, 0.05, 0.2, 0.5, 2, and 5 mg/kg, respectively (one more animal from 0.05 mg/kg died during anesthesia thus was only counted in hatchability). Three to five viable hatchling chickens were included for the hatchling cardiac morphological/functional assessments and immunohistochemistry. Three independent samples per group (excluding 0.05 or 5 mg/kg groups) were included for western blotting.

### Materials

PM2.5 from the 2016–2017 winter air of Beijing was acquired from a previous study (Chen S. et al., 2019). Briefly, the 24 h PM2.5 samples were collected with high-volume air samplers (Thermo, Waltham, MA, US) at a flow rate of 1.05 m^3^/min. Primary antibody against NF-kb p65 was purchased from Abcam (ab16502, Shanghai, China). Antibodies against iNOS and MMP9 were purchased from Bioss (bs-0162R for iNOS and bs-4593R for MMP9, Beijing, China). Antibody against GAPDH was purchased from ZSGB-BIO (TA-08, Beijing, China). Hematoxylin-eosin staining kit was purchased from Beyotime (C0105, Beijing, China). Immunohistochemistry kit was purchased from ZSGB-BIO (SP-9001, Beijing, China). Other general laboratory supplies are of the highest grade obtainable.

### Air-Cell Injection

Air-cell injection was performed on fertile chicken eggs prior to development (embryonic day zero, ED0) as described in Jiang et al. (2012) with slight modifications. The original method was used for the injection of perfluorooctanoic acid, which is adopted for PM2.5 in the current study. Briefly, the PM2.5 particles were thoroughly mixed with sunflower oil by vortexing vigorously for 30 seconds. The concentrations for the dosing suspension were: 0.5, 2, 5, 20, and 50 mg/ml PM2.5 in sunflower oil for final doses of 0.05, 0.2, 0.5, 2, and 5 mg/egg kg PM2.5, respectively. Control animals received vehicle injection (sunflower oil). It had been proven that the sunflower oil had no remarkable effects regarding to the cardiovascular development in chicken embryos (Jiang et al., 2012).The doses of PM2.5 is comparable to previous similar studies performed in rodents (Wang Z. et al., 2019). However, to the best of our knowledge, no similar avian studies were reported previously. Yang et al. mentioned that the ambient levels of particulate matters in chicken houses located at Shandong, China were 114 to 230 μg/m^3^, from which the estimated real-life daily exposure to particulate matter would be approximately 0.1–0.2 mg/kg/day in chickens at Shandong, China (Yang W. et al., 2018). Such doses were covered in the current study. Regarding to the environmental levels, it had been reported that the particulate matter levels ranged from 80–90 ug/m^3^ in the 2017 winter at Shijiazhuang, China (Li D. et al., 2019), while a mean level of 60.64 ug/m3 was observed in China from 2005–2016 (He et al., 2017). Although it is difficult to accurately estimate the total exposure burden per body weight from just the ambient concentrations, the actual exposure levels were likely at the lower ends of the doses used in the current study. The air cell area was cleaned with 75% ethanol briefly, a hole with diameter of approximately 1 mm was drilled with a metal probe, the dosing suspension was then injected into the air cell area at 0.1 μl/g (egg weight). The dosing suspension was vortexed for at least ten seconds immediately prior to every injection. After the injection, the hole was sealed with melted paraffin.

### Egg Incubation

Injected eggs were incubated in a Keyu incubator (Dezhou, China) as previously described (Lv et al., 2018). The incubation temperature and humidity were automatically controlled by the incubator. The incubation temperature was set at 37.9 degree Celsius at the beginning of incubation and gradually decreased to 37.1 degree Celsius as incubation continued, while humidity increased from 50 to 70%. Eggs were turned every three hours until ED19. Prior to hatch, externally pipped eggs were moved to individual hatch boxes, and hatched chickens were kept in a warm box with water supplied until the experiments were performed. This work has received approval for research ethics from the Institutional Animal Care and Use Committee (IACUC) of Qingdao University.

### Electrocardiography

Within 24 h post hatch, hatchling chickens were anesthetized with 33 mg/kg pentobarbital *via* intraperitoneal injection and then subjected to electrocardiography as previously described (Lv et al., 2018). Briefly, two electrodes were embedded on both sides of the chest subcutaneously, and then connected to a BL-420E+ instrument (Taimeng, Sichuan, China). Electrocardiography were recorded and the heart rates were calculated with R-R intervals with the following equation: Heart rate (beats per minute) = 60/R-R interval (second).

### Histological Assessments

For the ED4 chicken embryo, the whole embryos were removed from the eggs and directly visualized under a dissection microscope (Olympus SXZ9, Tokyo, Japan). The areas of heart of the embryos was quantified with ImageJ (NIH, US). Please refer to **Supplementary Figure 1** for a demonstration of the measurements.

For the hatchling chickens, animals were anesthetized and quickly decapitated. Heart tissues were carefully dissected out, rinsed in cold phosphate buffered saline (PBS), fixed in PBS-buffered 4% formaldehyde for 24 h, and then embedded with paraffin. Embedded tissues were then sectioned with a Leica RM2016 microtome at six μm thickness. Sections were then subjected to hematoxylin and eosin (HE) staining following instructions from manufacturer (Beyotime, Beijing, China), or immunohistochemistry (ZSGB-Bio SP-9001, Beijing, China) as previously described (Lv et al., 2018). Briefly, the sections were firstly deparaffinized with xylene, antigen-retrieved with citrate buffer, quenched with hydrogen peroxide, blocked with goat serum, probed with primary antibodies (1:1,000) or PBS as negative control, and then visualized with HRP-conjugated secondary antibody (Goat anti rabbit), streptavidin working solution and DAB working solution (Solarbio, Beijing, China). Resulting sections were briefly counterstained with hematoxylin and visualized with a dissection microscope (Phoenix SMZ180, Jiangxi, China). HE stained heart pictures were quantified as described in Jiang et al. for the right ventricular wall thickness. Immunohistochemistry pictures were quantified with ImageJ for the positive staining area ratio.

### Western Blotting

Hatchling chickens were anesthetized and quickly decapitated. Heart tissues were carefully dissected out and then homogenized in radio immunoprecipitation assay (RIPA) buffer (Beyotime, Beijing, China) with 1 mM PMSF (Solarbio, Beijing, China) and 2 μg/ml aproteinin (Solarbio, Beijing China) added. Western blotting was performed as described previously (Jiang et al., 2020). The protein concentrations of resulting samples were determined with BCA assays (Beyotime, Beijing, China) and adjusted with PBS. The samples were then subjected to electrophoresis, transferred to polyvinyldene fluoride (PVDF) membranes, blocked with non-fat milk, probed with iNOS, MMP9 or GAPDH primary antibodies (1:1,000 for iNOS/MMP9 and 1:5,000 for GAPDH) and corresponding secondary antibodies (1:5,000 HRP conjugated Anti-rabbit or mouse antibodies), visualized with a Fusion Solo S (Vilber Lourmat, Collégien, France) and quantified with ImageJ (NIH, US) software. Three independent samples were assessed per group.

### Lactate Dehydrogenase Activities Assay

The lactate dehydrogenase (LDH) activities in hatchling chicken serum were assessed with a commercially available kit (A020-2-2, Jiancheng, Nanjing, China) following manufacturer’s instructions.

### Superoxide Dismutase Activities Assay

The superoxide dismutase (SOD) activities in hatchling chicken serum were assessed with a commercially available kit (A001-3-2, Jiancheng, Nanjing, China) following manufactuer’s instructions.

### Statistical Analysis

Data were represented as mean ± standard derivation. Statistical analysis was performed with SPSS 17.0. One-way analysis of variance (ANOVA) was used to assess differences among groups. When ANOVA detected significant results, post-hoc tests (least significant difference test) were used to compare differences between groups. Results were considered statistically significant when P < 0.05.

## Results

### Particulate Matter 2.5 Component Analysis

To confirm the component of the PM2.5 used in the current study, component analysis was performed. The collection and identification method of the PM2.5 was as described in Chen S. et al. (2019). The polycyclic aromatic hydrocarbons (PAHs) was estimated at 67.273 ng/m^3^, and the alkyl PAHs was estimated at 16.406 ng/m^3^ (Chen S. et al., 2019). Additionally, the water extraction of the PM2.5 was analyzed with inductively coupled plasma mass spectrometry (ICP-MS; ELEMENT2;221 Thermo Finnigan, San Jose, CA, USA), and the results was reported in **Table 1**.

**Table 1:** The concentrations of metals and ions bound to PM2.5.

Parameters	(ng/m^3^)
**Metal elements**	
Li	2.434
Be	0.017
Na	1040.235
Mg	65.677
Al	67.498
K	1624.904
Ca	266.734
Ti	0.707
V	17.142
Cr	5.063
Mn	43.747
Fe	213.251
Co	0.271
Ni	6.263
Cu	28.747
Zn	481.346
Ga	1.128
As	15.747
Se	4.662
Rb	5.183
Sr	1.831
Y	0.059
Zr	0.265
Nb	0.011
Mo	1.266
Pd	0.003
Ag	BDL^a^
Cd	2.189
Sn	2.469
Sb	4.363
Te	0.210
Ba	5.204
Ta	BDL^a^
W	0.442
Tl	0.488
Pb	37.403
**Total metals**	3915.461
**Anions**	
F^-^	51.486
Cl^-^	549.033
SO_4_^2-^	21217.029
NO_3_^-^	1833.796
PO_4_^3-^	71.513
NO_2_^-^	5.028
Br^-^	43.481
**Total anions**	23771.366

a. BDL, below detection limit.

### Areas of ED4 Chicken Embryo Heart

**Figure 1:**
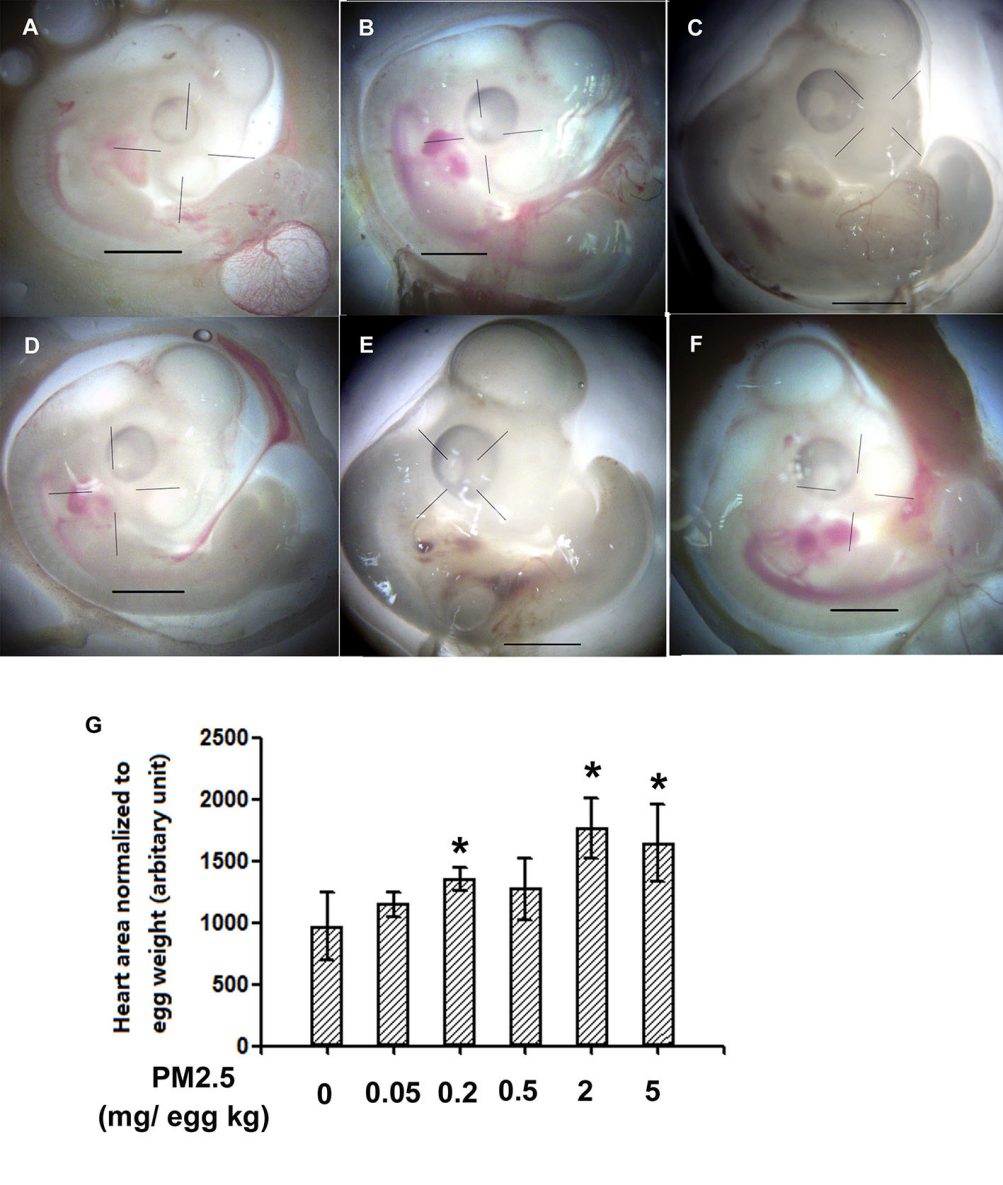
Morphological assessment of areas of embryonic day 4 chicken embryo heart. Fertile chicken eggs were air-cell injected with 0, 0.05, 0.2, 0.5, 2, and 5 mg/kg PM2.5 in sunflower oil, and incubated until embryonic day 4. Embryos were then taken out of the shell and directly visualized with a microscope (Olympus SXZ9, Tokyo, Japan). ImageJ was used to quantify the area of the hearts. N=3–5 per group. *: statistically different from control (P < 0.05). Scale bars represent 1,000 μm. **(A)** Representative photo of control ED4 embryo. **(B)** Representative photo of ED4 embryos exposed to 0.05 mg/kg PM2.5. **(C)** Representative photo of ED4 embryos exposed to 0.2 mg/kg PM2.5. **(D)** Representative photo of ED4 embryos exposed to 0. 5 mg/kg PM2.5. **(E)** Representative photo of ED4 embryos exposed to 2 mg/kg PM2.5. **(F)** Representative photo of ED4 embryos exposed to 5 mg/kg PM2.5. **(G)** Quantification of the areas of heart.

To investigate whether developmental exposure to PM2.5 may affect early heart development in chicken embryos, the areas of heart in ED4 chicken embryo were measured and analyzed. Significantly increased areas of heart were observed in 0.2, 2, and 5 mg/kg PM2.5-exposed ED4 chicken embryos, resulting in 39.9, 82.5, and 70.2% increase relative to control embryo hearts, respectively (**Figure 1**).

### General Parameters of Hatchling Chickens

**Figure 2:**
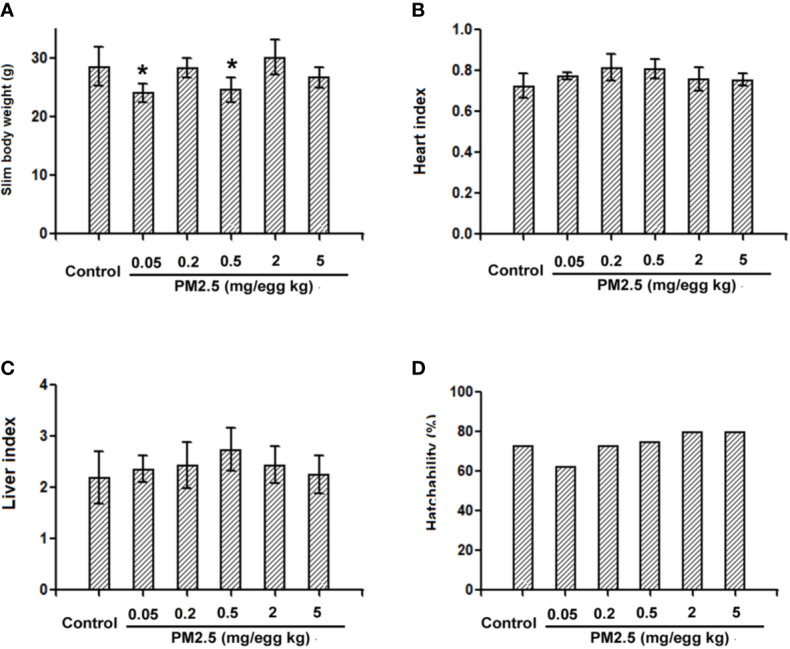
General parameters of hatchling chickens. Fertile chicken eggs were air-cell injected with 0, 0.05, 0.2, 0.5, 2, and 5 mg/kg PM2.5 in sunflower oil, and incubated until hatch. Within 24 h post hatch, hatchling chickens were anaesthetized with 33 mg/kg pentobarbital *via* intraperitoneal injection, weighed and sacrificed, yolks, hearts and livers were collected and weighed, slim body weight, heart index, liver index and hatchability were calculated. N=4–8 per group. *: statistically different from control group (P < 0.05). **(A)** Slim body weight of the hatchling chickens [(Body weight – yolk weight)*100%]. **(B)** Heart index of the hatchling chickens [(heart weight/slim body weight)*100%]. **(C)** Liver index of the hatchling chickens [(liver weight/slim body weight)*100%]. **(D)** Hatchability of the hatchling chickens (number of successful hatchlings/total number of embryos reached hatching stage). Note: one animal from 0.05 mg/kg group died during anesthesia thus was not counted in slim body weight, heart index or liver index.

To confirm whether overt toxicities were present following developmental exposure to PM2.5, general parameters were analyzed and reported in **Figure 2**. Decreased slim body weight (body weight minus yolk weight) was observed in the hatchling chickens developmentally exposed to 0.05 and 0.5 mg/kg PM2.5 (18.1 and 16.0%, respectively). No statistically changes were observed in other endpoints, including the heart index (heart weight normalized to slim body weight for the specific cardiac effects), liver index (liver weight normalized to slim body weight), and hatchability (number of successful hatches divided by total number of embryos reached hatchling stage) (**Figure 2**).

### Hatchling Chicken Heart Rates

**Figure 3:**
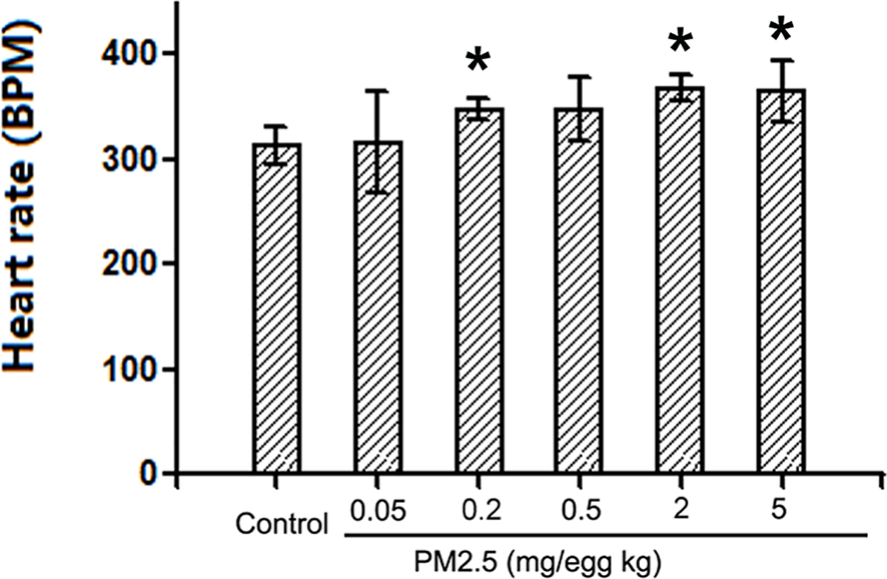
Heart rates of hatchling chickens. Fertile chicken eggs were air-cell injected with 0, 0.05, 0.2, 0.5, 2, and 5 mg/kg PM2.5 in sunflower oil, and incubated until hatch. Hatchling chickens were anaesthetized with 33 mg/kg pentobarbital *via* intraperitoneal injection, and subjected to electrocardiography with the BL-420E+ (Taimeng, Chengdu, China). Heart rates were calculated with the equation: 60/R-R interval. N=3–5 per group. *: statistically different from control (P < 0.05).

To assess the potential cardiac functional changes following developmental exposure to PM2.5, electrocardiography was used to assess the heart rates of hatchling chickens. At the higher doses tested (0.2, 2, and 5 mg/kg), PM2.5 developmental exposures led to significantly elevated heart rates relative to control, resulting in 11.1, 17.4, and 16.6% increase, respectively (**Figure 3**).

### Histological Assessment of Hatchling Chicken Heart Right Ventricular Wall Thickness

**Figure 4:**
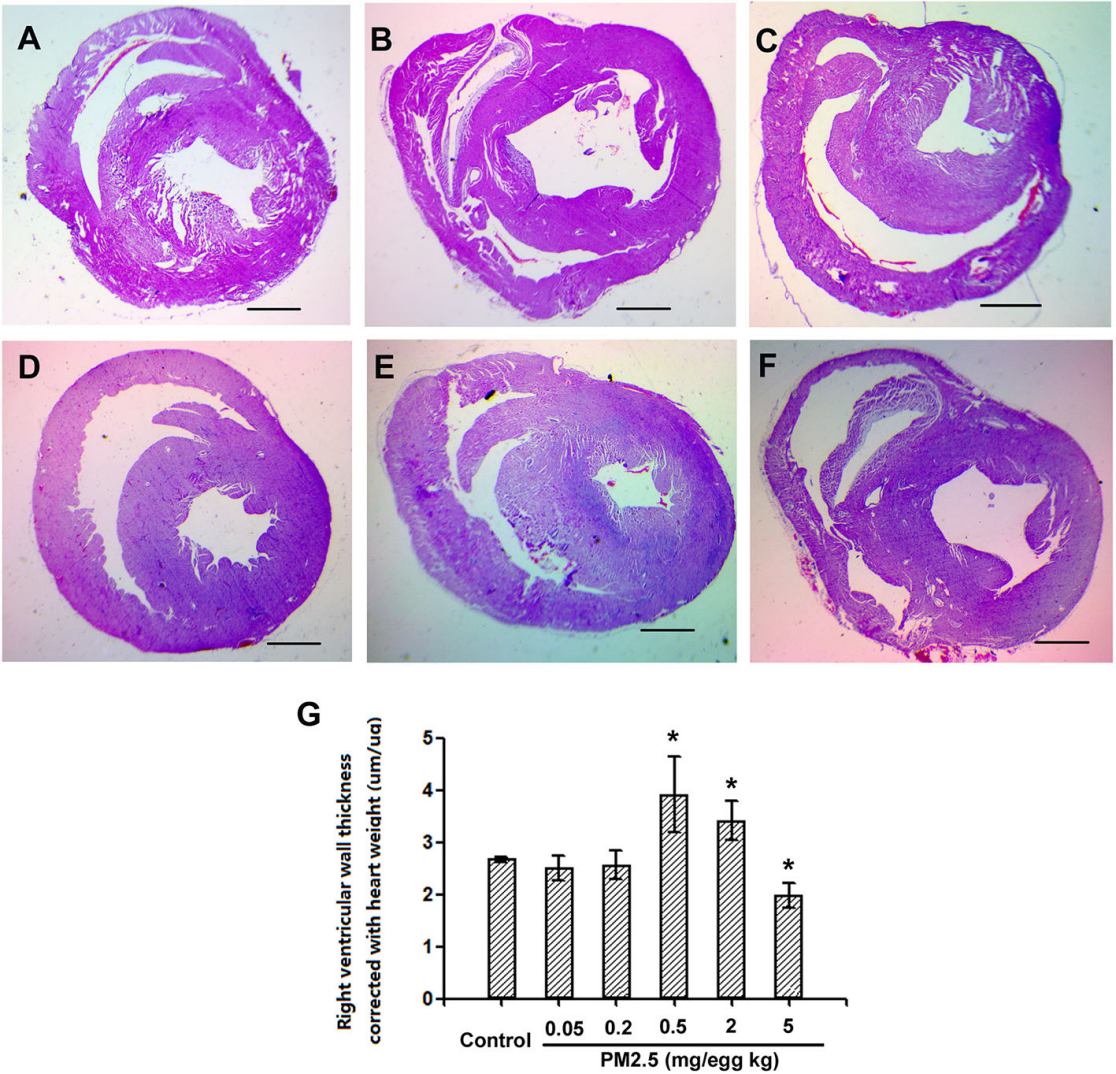
Histological assessment of hatchling chicken hearts. Fertile chicken eggs were air-cell injected with 0, 0.05, 0.2, 0.5, 2, and 5 mg/kg PM2.5 in sunflower oil, and incubated until hatch. Animals were sacrificed and hearts were collected, fixed in 4% formaldehyde for 24 h, and embedded in paraffin. Six μm sections were made and stained with hematoxylin and eosin, resulting slides were visualized with a microscope (Olympus SXZ9, Tokyo, Japan), and measured for the right ventricular wall thickness. N=3 per group. *: statistically different from control (P < 0.05). Scale bars represent 1,000 μm. **(A)** Representative picture of hearts from control animals. **(B)** Representative picture of hearts from animals exposed to 0.05 mg/kg PM2.5. **(C)** Representative picture of hearts from animals exposed to 0.2 mg/kg PM2.5. **(D)** Representative picture of hearts from animals exposed to 0.5 mg/kg PM2.5. **(E)** Representative picture of hearts from animals exposed to 2 mg/kg PM2.5. **(F)** Representative picture of hearts from animals exposed to 5 mg/kg PM2.5. **(G)** Quantification of right ventricular wall thickness.

To assess the potential cardiac morphological changes following developmental exposure to PM2.5, the right ventricular wall thickness was assessed with histological methods. While no significant changes were observed following 0.05 or 0.2 mg/kg PM2.5 developmental exposure, 0.5 and 2 mg/kg PM2.5 exposure led to significantly elevated right ventricular wall thickness (46.2 and 38.6% comparing to control, respectively). Interestingly, the highest dose tested (5 mg/kg) resulted in remarkably decreased right ventricular wall thickness (26.2% relative to control) (**Figure 4**).

### Immunohistochemistry for NF-kb p65 in Hatchling Chicken Hearts

**Figure 5:**
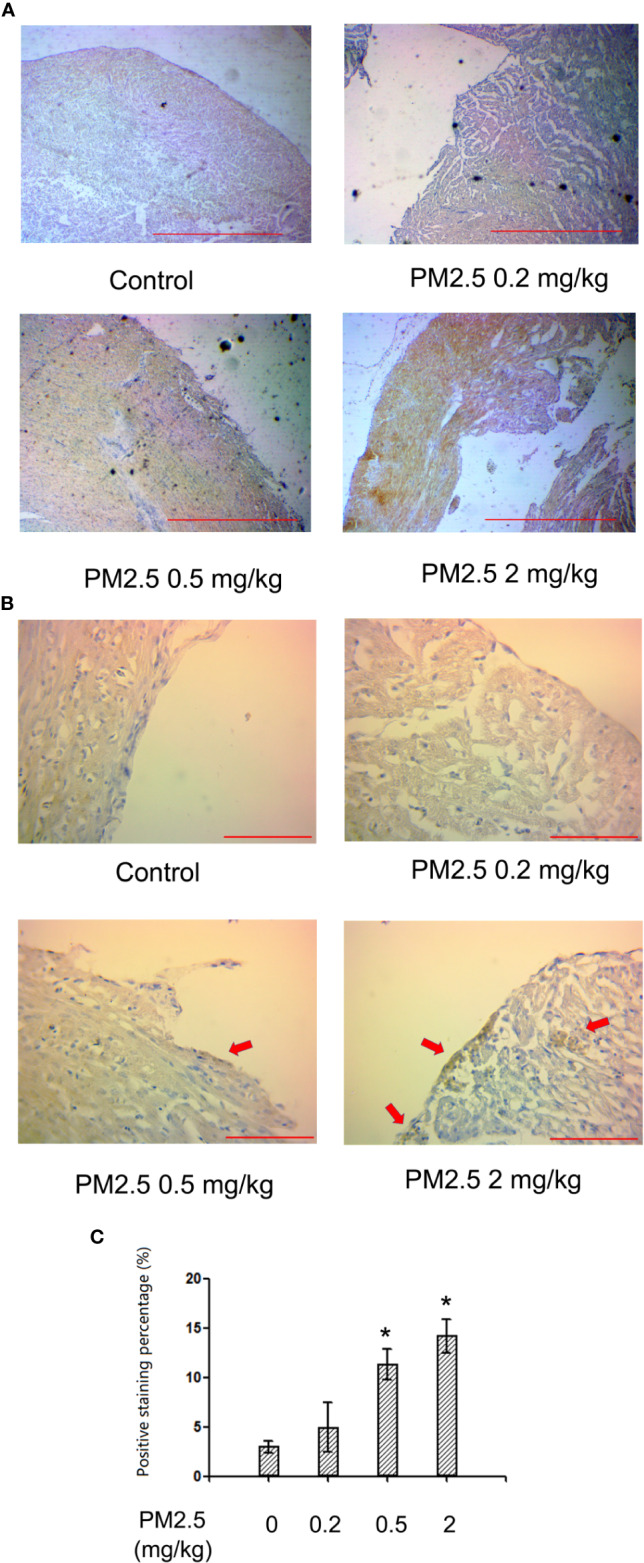
Immunohistochemistry for NF-kb p65 in hatchling chicken hearts. Fertile chicken eggs were air-cell injected with 0, 0.2, 0.5, and 2 mg/kg PM2.5 in sunflower oil, and incubated until hatch. Animals were sacrificed and hearts were collected, fixed in 4% formaldehyde for 24 h, and embedded in paraffin. Six μm sections were made and subjected to immunohistochemistry for NF-kb p65. **(A)** Representative low magnitude pictures. Scale bars represent 600 μm. **(B)** Representative high magnitude pictures. Arrows indicate the positive staining areas. Scale bars represent 70 μm. **(C)** Quantification results from the high magnitude pictures. N=3 per group. *: statistically different from control (P < 0.05).

To confirm whether NF-kb p65 is involved in PM2.5-exposure induced developmental cardiotoxicity in chicken embryo, immunohistochemistry for NF-kb p65 was performed on the hatchling heart sections. While no significant changes were observed in 0.2 mg/kg, significantly higher levels of positively stained cells were present in tissues from 0.5 and 2 mg/kg PM2.5-exposed animals. Quantification revealed a relative change of 278.8 and 375.9%, respectively (**Figure 5**).

### Western Blotting for iNOS and MMP9 Expression Levels in Hatchling Chicken Hearts

**Figure 6:**
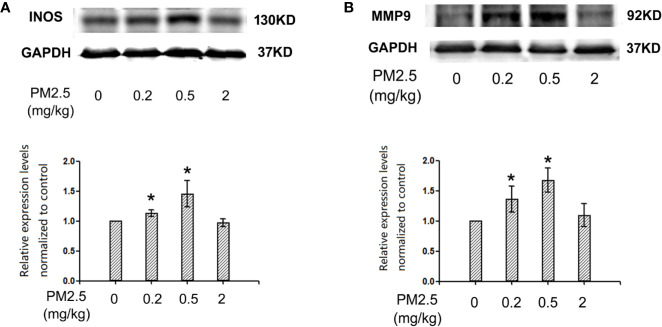
Western blotting for iNOS and MMP9 in hatchling chicken hearts. Fertile chicken eggs were air-cell injected with 0, 0.2, 0.5, and 2 mg/kg PM2.5 in sunflower oil, and incubated until hatch. Animals were sacrificed and hearts were collected, protein samples were extracted and subjected to western blotting. Results were semi-quantified with ImageJ by firstly normalizing to the internal controls (GAPDH), then normalizing to the control sample on the same gel. N=3 per group. *: statistically different from control (P < 0.05). **(A)** Representative images and semi-quantifications for iNOS. **(B)** Representative images and semi-quantifications for MMP9.

To assess the potential changes of major inflammation/cardiac remodeling mediators, iNOS and MMP9, western blotting was performed with heart protein extracts. The results indicated similar results of iNOS and MMP9 expression levels in hatchling chicken heart: significantly elevated expression levels in 0.2 and 0.5 mg/kg groups, but no remarkable changes in 2 mg/kg groups. For iNOS, the percentage changes in 0.2 and 0.5 mg/kg groups were 13.2 and 45.5%, respectively. For MMP9, the percentage changes in 0.2 and 0.5 mg/kg groups were 36.2 and 67.5%, respectively (**Figure 6**).

### Lactate Dehydrogenase and Superoxidase Dismutase Activities in Hatchling Chicken Serum

**Figure 7:**
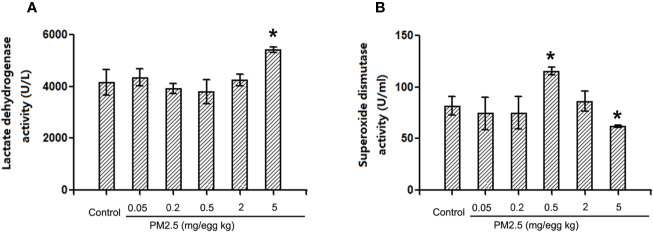
Lactate dehydrogenase and superoxide dismutase activities in hatchling chicken serum. Fertile chicken eggs were air-cell injected with 0, 0.2, 0.5, and 2 mg/kg PM2.5 in sunflower oil, and incubated until hatch. Animals were sacrificed, serum were collected and subjected to LDH and SOD activities assays. N=3 per group. *: statistically different from control (P < 0.05). **(A)** Quantification of lactate dehydrogenase activities. **(B)** Quantification of superoxide dismutase activities.

To assess the potential direct myocardium damage and oxidative stress, LDH activities and SOD activities were assessed in hatchling chicken serum, respectively. The results indicated that LDH activities were not affected in hatchling chicken serum following 0.05, 0.2, or 2 mg/kg PM 2.5 exposure, but significantly increased following 5 mg/kg PM2.5 exposure (30.4% relative to control, **Figure 7A**). On the other hand, no remarkable changes were observed in SOD activities following 0.05, 0.2, or 2 mg/kg PM2.5 exposure, but significantly increased SOD activities were observed in serums of hatchling chickens exposed to 0.5 mg/kg PM2.5 (41.5% relative to control), and remarkably decreased SOD activities were observed in those exposed to 5 mg/kg PM2.5 (24.1% relative to control, **Figure 7B**).

## Discussion

PM2.5 exposure affects a large population among the world. While its negative health effects are currently being extensively investigated, the developmental toxicities received relatively less attention. Moreover, the developmental cardiotoxicity was even less investigated, while heart development is one of the most important early events in embryo development (Astrof et al., 2007) and potentially affects cardiovascular health after birth and even in adulthood (Klattenhoff et al., 2013). The current study utilized the classical chicken embryo model to investigate the potential developmental cardiotoxicity induced by PM2.5 exposure, revealing potential effects exerted by developmental exposure to PM2.5 and the underlying molecular mechanism. While many still remain to be elucidated, our data suggests that developmental exposure to PM2.5 has the potential to interfere with heart development, and is associated with inflammatory responses.

The real-world PM2.5 exposure occurs by inhalation, while in utero exposure is possible since PMs may pass the placenta barrier (Teng et al., 2016). In laboratory, it is generally difficult to expose animals with accurate doses according to the ambient concentrations, and alternative exposure methods, such as intratracheal instillation or direct exposure are commonly used. One study performed in rats used 9 and 24 mg/kg PM exposure *via* intratracheal instillation for seven weeks (Wei et al., 2018). Another study exposed zebrafish embryos with up to 900 μg/ml PM2.5 in water for 120 h (Zhang Y. et al., 2018). In the current study, 0, 0.05, 0.2, 0.5, 2, and 5 mg/kg PM2.5 were exposed to fertile chicken eggs as a one-time air cell injection, which is comparable to previous studies. From He et al. (2017), the average particulate matter level in China from 2005–2016 was 60.64 ug/m^3^. The lowest dose (0.05 mg/kg) used in the study may be roughly translated to approximately 175 hours’ exposure at that level, while the highest dose used (5 mg/kg) roughly equals 2 years’ exposure. Since chicken eggs are closed systems, the injected PM2.5 stays in the shell for the whole embryonic development period (21 days). The lowest and highest doses (0.05 and 5 mg/kg) were not included in the immunohistochemistry and western blotting experiments, since 0.05 mg/kg induced little effects, while 5 mg/kg seemed to start to induce overt toxicity from the histological data.

PM2.5 exposure occurs almost ubiquitously in areas with air pollution, resulting in definite exposure to developing embryo/fetus in these areas, thus the potential developmental toxicity is a concern. Many toxic components, such as the heavy metals and PAHs are present in PM, which are capable of exerting developmental toxicities (Shankar et al., 2019; Zhao et al., 2020). Epidemiological studies revealed that developmental exposure to PM resulted in increased risk of asthma (Kravitz-Wirtz et al., 2018), decreased airway antimicrobial activity (Zhang et al., 2019), low birth weight (Wang et al., 2019b), and pregnancy loss (Xue et al., 2019).

In the current study, lower body weight was observed in hatchling chickens, which is consistent with the epidemiological results. Regarding to the heart, although many studies focused on the cardiovascular effects following PM exposure in general population (Hamanaka and Mutlu, 2018), no epidemiological reports have associated cardiovascular effects with developmental exposure to PM to the best of our knowledge. Nevertheless, several laboratory studies reported developmental cardiotoxicity in zebra fish model, and associated the mechanism with AhR signaling (Yue et al., 2017; Chen T. et al., 2019). In these studies, abnormal heart structure was observed following PM exposure, suggesting that PM2.5 has the potential to interference with cardiac morphology, which may be explained by the fact that both metals and PAHs in PM are capable of inducing developmental cardiotoxicity (Jayasundara et al., 2015; Liu et al., 2018). In the current study, morphological changes in chicken embryo/hatchling chicken hearts were observed following PM2.5 developmental exposure, confirming the previous studies performed with zebra fish embryos. While the heart index did not change in hatchling chickens, suggesting that exposure to PM2.5 did not directly retard heart development, thickened right ventricular wall thickness was observed, which was a typical change following pulmonary damage (Vonk Noordegraaf et al., 2017). Since right ventricle was typically associated with cardiopulmonary effects, observed changes suggested that PM exposure may target pulmonary system and right ventricle. Notably, different doses of PM exposure resulted in different responses. While doses equal or lower than 0.2 mg/kg did not induce significant changes, 0.5 and 2 mg/kg exposure to PM resulted in remarkably thicker right ventricular walls. Interestingly, 5 mg/kg exposure did not further increase the right ventricular wall thickness, instead, the thickness significantly decreased, even thinner than those of the control animals. Such effects may be the results of overt toxicity at higher doses, as demonstrated by histological results. Additionally, elevated heart rate was observed with electrocardiography, which had been associated with myocardial injury and cardiac dysfunction (Abbott et al., 2018; Grande et al., 2018). It was also worth noting that the altered heart morphology and function are consistent with the teratogenic effects reported by Ejaz, S. and Massarsky, A., suggesting that the particulate matter used in the current study has teratogenic potential at the doses tested (Ejaz et al., 2009; Massarsky et al., 2015).

NF-kb signaling pathway is involved in tissue damage, especially inflammatory responses (Zhang et al., 2017; Song et al., 2018). It had been reported that NF-kb participates in PM2.5-induced toxicities, including atherosclerosis (Geng et al., 2019), pulmonary toxicity (Shi et al., 2019), and general inflammation (Fukatsu et al., 2018; Ryu et al., 2019). Inflammation plays important role in general cardiac hypertrophy (Li H. et al., 2019b; Li Q. et al., 2019) as well as particulate matter-induced cardiotoxicity (Luo et al., 2019). In the current study, elevated NF-kb expression levels were observed in exposed animal hearts, indicating the involvement of NF-kb and potentially inflammation in PM2.5-induced developmental cardiotoxicity. Interestingly, cumulated positive stains in clusters were observed in immunohistochemistry, suggesting that the inflammation tend to occur in relatively localized locations rather than diffused throughout the whole organ. This phenomenon will be further investigated.

Oxidative stress plays important roles in particulate matter-induced cardiotoxicities (Rao et al., 2018). Several studies associated oxidative stress with cardiac dysfunction (Qin et al., 2018), cardiac hypertrophy (Yue et al., 2019), and cardiac fibrosis (Gao et al., 2020). One important marker and potentially contributor of oxidative stress is iNOS, which is an inducible enzyme, producing excessive amounts of NO in case of inflammation or oxidative stress elevation. On the other hand, MMP9 participates in the degradation of the extracellular matrix during tissue remodeling (Lee et al., 2012; Wilmes et al., 2020). Both iNOS and MMP9 had been associated with PM2.5-induced toxicities frequently (Li et al., 2015; Wang et al., 2016; Yang B. et al., 2018; Zhang H. et al., 2018). iNOS is typically induced in response to inflammation, thus might participate in the developmental cardiotoxicity observed in the current study. MMP9 is an important factor in cardiac remodeling (Jenke et al., 2017), and might partially explain the morphological changes in the hearts following PM2.5 exposure. Moreover, iNOS and MMP9 are closely related to each other, often involved in toxicities together and had been both reported to be regulated by NF-kb (Yu J. et al., 2019). In the current study, enhanced expression of both iNOS and MMP9 were observed following PM exposure in hatchling chicken hearts, suggesting involvement of iNOS and MMP9 in the observed developmental cardiotoxicity, and further confirms the involvement of inflammation/oxidative stress. Direct measurement of LDH and SOD activities in serum also provided evidence that higher doses of PM exposure induced myocardium damage and oxidative stress. Similar expression patterns indicated that these two are likely to be regulated together. Interestingly, the highest dose tested for iNOS and MMP9 (2 mg/kg) did not have significant impact, while the two lower doses did (0.2 and 0.5 mg/kg). Since similar pattern (higher doses generate different responses from lower ones) were also observed in slim body weight and histopathology data, the effect is unlikely caused by chance. Actually, many toxicants featured such non-linear dose-response curves, sometimes are known as hormesis (Teeguarden et al., 2000), usually resulting from low-dose exposure-induced defense/compensation. It is also worth noting that the above-mentioned immunohistochemistry results indicated no such trends for NF-kb expression in heart tissues. A possible explanation is that some other unknown regulatory/defensive factors started to take effect at higher doses, suppressing the expression of iNOS and MMP9, while the relatively up-stream NF-kb was not covered by those unknown factors. Whether the lower doses of effect can be interpreted as protective/hormesis remain to be elucidated. In summary, the NF-kb-iNOS/MMP9 pathway is associated with PM2.5-induced developmental cardiotoxicity in chicken embryo. To further elucidate the mechanisms, further investigations on this specific pathway is guaranteed.

## Conclusions

In the current study, developmental exposure to PM2.5 particles induced morphological and functional changes in the hearts of developing chicken embryo and hatchling chickens, suggesting developmental cardiotoxicity. Mechanistic investigations associated NF-kb p65, iNOS, and MMP9 with the observed endpoints. Further investigations regarding to such effects is necessary.

## Data Availability Statement

The materials described in the article, including all relevant raw data, will be freely available to any scientist wishing to use them for non-commercial purposes, and the data used in the current study can be obtained from the corresponding author on a reasonable request.

## Ethics Statement

The animal study was reviewed and approved by Institutional Animal Care and Use Committee (IACUC) of Qingdao University.

## Author Contributions

YZ and QJ designed the study. QJ and CZ performed the data analysis and drafted the manuscript. SC and DL assisted the manuscript writing. QJ, SC, and LC performed the statistical analysis. SC and DL performed the particulate matter 2.5 content analysis. QJ, CZ, LS, and NL performed animals’ examinations and collected data. QJ and YC performed the histopathological assessments. LS and NL performed western blotting experiments. YZ reviewed and edited the manuscript. All authors read and approved the final manuscript.

## Funding

This work was supported by National Natural Science Foundation of China (Grant No. 91643203, 91543208, 81872591, 81502835).

## Conflict of Interest

The authors declare that the research was conducted in the absence of any commercial or financial relationships that could be construed as a potential conflict of interest.

## Supplementary Material

Supplementary Figure 1 Measurement method for the areas of heart in embryonic day four (ED4) chicken embryos. The blue circle represents the area of heart, whose area was then measured with ImageJ (NIH, US).
