# Supplementary figures and images for: Particulate Matter 2.5 Induced Developmental Cardiotoxicity in Chicken Embryo and Hatchling
Source: Front Pharmacol. 2020 Jun 5;11:841. doi: 10.3389/fphar.2020.00841 (PMC7289969; doi:10.3389/fphar.2020.00841)

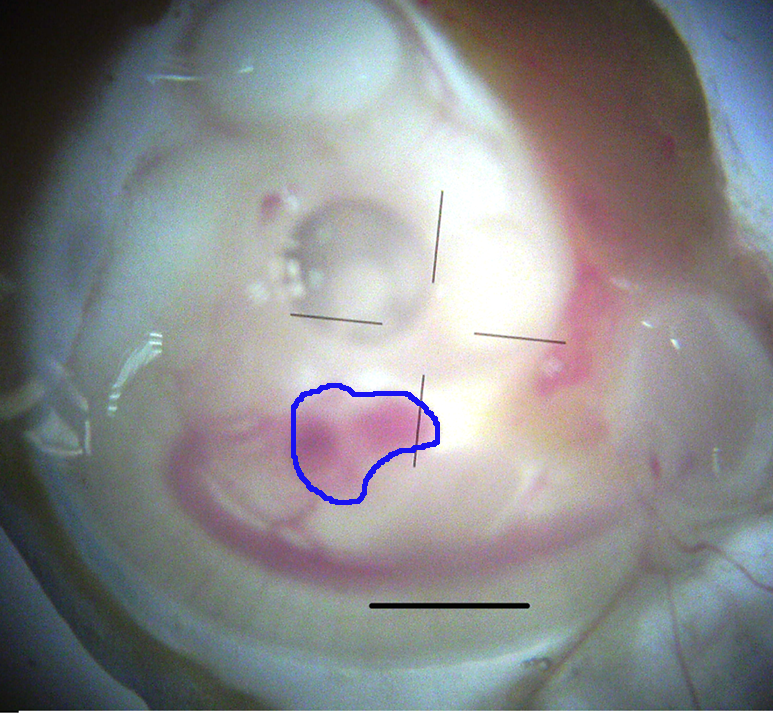

Supplement: Supplementary Figure 1 — Measurement method for the areas of heart in embryonic day four (ED4) chicken embryos. The blue circle represents the area of heart, whose area was then measured with ImageJ (NIH, US). [file Image_1.tif]
